# Sensitive and accurate determination of oil-soluble and water-soluble organosulfur compounds in garlic matrix using reversed phase-high performance liquid chromatography

**DOI:** 10.55730/1300-0527.3658

**Published:** 2024-03-01

**Authors:** Ümmügülsüm POLAT KORKUNÇ, Buse Tuğba ZAMAN, Sezgin BAKIRDERE, Emine KARAKUŞ

**Affiliations:** 1 Department of Chemistry, Faculty of Arts and Science, Yıldız Technical University, İstanbul, Turkiye; 2 Turkish Academy of Sciences (TÜBA), Ankara, Turkiye

**Keywords:** Organosulfur compounds, high-performance liquid chromatography, garlic, extraction

## Abstract

Garlic is in the family Liliaceae and has many different constituents such as organic sulfur compounds, amino acids, carbohydrates, minerals, and vitamins. In this work, a simple, sensitive, and accurate analytical method was developed for the determination of selected organosulfur compounds (OSCs) in garlic bulbs using reversed phase-high performance liquid chromatography (RP-HPLC). Oil-soluble and water-soluble OSCs were extracted from the garlic samples via acetonitrile and deionized water, respectively. The OSCs were separated on a Phenomenex C18 (250 mm, 4 mm, 5mm) column and the monitoring was performed with a UV detector at 220 nm. An isocratic mobile phase comprising of 0.10 M trifluoroacetic acid (TFA) in 85% acetonitrile (ACN) and 0.10 M TFA in distilled water (DW) (90: 10% v/v) was used to elute the analytes. Under optimum experimental conditions, the limits of detection (LOD) for the analytes were calculated in the range of 0.09 to 0.17 mg/kg. For the garlic sample extracts analyzed under optimal instrumental conditions, DAS (diallyl sulfide), DADS (diallyl disulfide), and DATS (diallyl trisulfide) were detected in the ranges of 8.0 to 32.5 mg/kg, 20.4 to 67.3 mg/kg, and 60.7 to 356.6 mg/kg, respectively. Spiked recovery experiments were conducted on the garlic samples to confirm the method’s applicability and accuracy. The recovery results ranged between 39.0% and 90.9% for the garlic samples extracted with deionized water. The developed method is simple, precise, accurate, reliable, and time-effective for the determination of OSCs. Additionally, the green profile of the developed method was investigated by using AGREEprep software and the greenness score was recorded as 0.65, indicating that the method developed is environmentally friendly.

## 1. Introduction

Allium sativum (garlic) is a durable plant belonging to the Liliaceae family, which has been used over centuries for its biological and medical properties. Its benefits are thought to stem from the high abundance of organosulfur compounds (OSCs) in the vegetable [1–4]. OSCs are categorized into two groups: oil-soluble and water-soluble compounds. Recent studies have revealed that the benefits of garlic result from the presence of OSCs such as diallyl sulfide (DAS), diallyl disulfide (DADS) and diallyl trisulfide (DATS) [5]. It is believed that the number of sulfur atoms play an important role in the biological functions of OSCs in garlic [6]. DAS, which is an important component of garlic, has been demonstrated to have a potential chemopreventive effect against human cancers such as lung, skin, and colon [7]. Hydrogen peroxide easily oxidizes DADS to allicin, which can be hydrolyzed to yield DADS and DATS [8]. Due to its ability to elevate reduced glutathione’s intracellular concentration, DADS has been reported to have antioxidant effects [9]. Furthermore, it has also been reported that DATS exhibits considerable anticancer properties [10]. By activating apoptosis and stopping the continuation of abnormal cell cycles, DATS can restrict cancer cells’ proliferation and the formation of tumor xenografts [11]. In a study that investigated the effect of DADS and DATS on acute cardiac muscle disease caused by diabetes mellitus in mice, DATS was found to be more effective than DADS [12]. Among the organosulfur compounds, DATS was found to suppress the development of A375 cell tumors and prevent skin cancer by inhibiting DNA damage compared to DADS and DAS [13].

In the literature, these compounds have been qualitatively and quantitatively determined using ultraviolet-visible (UV-Vis) spectrophotometry [14], gas chromatography mass spectrometry (GC-MS) [15], selected ion flow tube mass spectrometry (SIFT-MS) [16], high performance thin layer chromatography (HPTLC) [17]. gas chromatography (GC) [18], high performance liquid chromatography (HPLC) [19], and liquid chromatography mass spectrometry (LC-MS) [20]. Among these instruments, HPLC is typically preferred because of its advantages including high repeatability, online detection, quick analysis, decreased handling, and a wide dynamic range [21].

The aim of this study was to determine the organosulfur compounds DAS, DADS, and DATS at trace levels in Kastamonu Taşköprü garlic samples by reverse phase high-performance liquid chromatography ultraviolet detection (RP-HPLC-UV). In the literature, no studies have been reported for the determination of trace amounts of allyl sulfide compounds in Kastamonu Taşköprü garlic based on oil- and water-soluble extractions.

## 2. Materials and methods

### 2.1 Instrumentation and chromatographic conditions

Determination of the OSCs was performed with Shimadzu (Kyoto, Japan) LC- 20AT HPLC coupled with an automatic sampler (SIL- 20A HT), a delivery pump, an oven (CTO- 10AS VP), and a UV-Vis detector (SPD- 20A). A Sigma- 3K30 model ultracentrifuge (Beckman Coulter, Inc., California, USA) was used for the separation of water- and oil-soluble extracts. Shimadzu UV2600 model UV-Vis spectrometer was used to determine maximum absorbance wavelength of the target analytes. Phenomenex C18 (250 mm, 4 mm, 5mm) (California, USA) and Zorbax ODS (250 mm, 4.6 mm, 5μm) columns (Agilent Technologies, Inc., California, USA) were used for chromatographic elution of the target analytes. The analytical wavelength, injection volume, and column temperature were respectively set at 220 nm, 20 μL, and 25 °C for all experiments. A mixture of trifluoroacetic acid (TFA) and acetonitrile (ACN) was employed as mobile phase with a flow rate of 1.0 mL/min. The mobile phase was prepared with a 90:10 (v/v) ratio of 0.10 M TFA (in 85% of ACN) to 0.10 M TFA in deionized water (DW) under isocratic conditions. The total run time was 8.0 min, and the retention times determined for the target analytes were 3.8 min for DAS, 4.2 min for DADS, and 5.1 min for DATS.

### 2.2 Reagents and chemicals

All chemicals and reagents used in all experiments of the study were of analytical grade. Pure standards of DAD, DADS, DATS, and glutathione were purchased from Sigma-Aldrich (St. Louis, MO, USA). The stock solutions were gravimetrically prepared at a concentration of 1000 mg/kg in acetonitrile. Deionized water was obtained from an Elga-Maxima Ultrapure Water System (ELGA LabWater, London, UK). HPLC grade ACN (>99%) and TFA (>99%) were respectively purchased from ISOLAB Laborgeräte GmbH (Eschau, Germany) and Merck (Darmstadt, Germany) and used for the preparation of the mobile phase.

### 2.3 Organosulfur compound extraction

The garlic samples (Allium sativum) were cultivated in Taşköprü (Kastamonu, Türkiye). The extraction of OSCs from the samples was carried out following a procedure reported in the literature [22]. The Kastamonu Taşköprü garlic bulbs were peeled and washed with distilled water. The OSCs were extracted by two successive extraction procedures for water-soluble and oil-soluble compounds, where the water-soluble extraction preceded the oil-soluble extraction. In this process, approximately 5 g of unspiked garlic cloves and 5 g of garlic cloves spiked with 200 mL of 100 mM glutathione were placed in test tubes and 10 mL of distilled water was added to each tube. Then, the garlic cloves were smashed for 10 min before the hot and cold extraction procedures. For the cold extraction procedure, the garlic samples were kept in a Dewar flask for 5.0 s, while the homogenates were placed in a 100 °C water bath for 15 min for the hot extraction. Moreover, the homogenates were centrifuged, and the yielded fractions (supernatant and pellets) were completely separated. The supernatant containing the water-soluble compounds was kept at −20 °C in a freezer. Afterwards, the remaining pellet (oil-soluble extraction) was incubated at room temperature in 10 mL of acetonitrile for 24 h. Then, the incubated oil-soluble extracts were centrifuged for 45 min at +4.0 °C and 9000 rpm. The water- and oil-soluble fractions were ultrafiltered at 4.0 °C through 0.45 μm filters. The ultrafiltered fractions were kept at −20 °C prior to identification and quantification by RP-HPLC-UV.

## 3. Result and discussion

**Figure 1:**
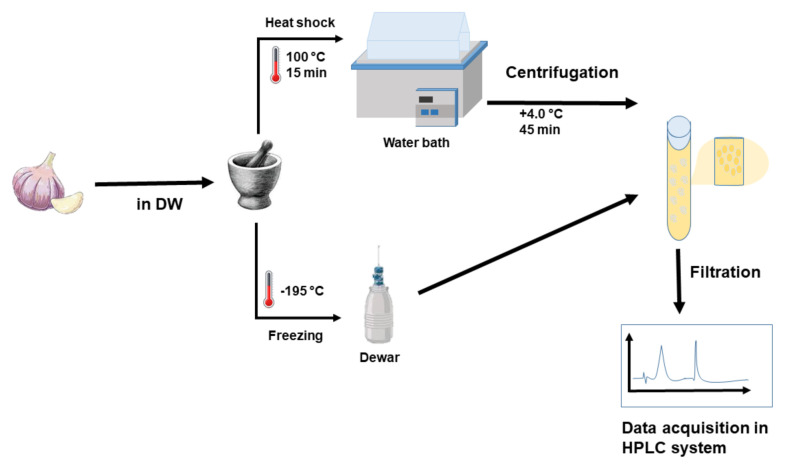
The experimental depiction of the developed method for OSCs determination in garlic samples by using RP-HPLC-UV system.

In order to obtain the most appropriate experimental conditions, parameters affecting the performance of the detection system were optimized. The parameters optimized were analytical column, mobile phase composition and ratio, wavelength, flow rate and injection volume. The experimental procedure is depicted in Figure 1.

### 3.1 Chromatographic separation of OSCs

All instrumental conditions and system parameters that affect determination efficiency were optimized to obtain accurate and precise quantification results for the analytes. The retention times of the analytes were determined by qualitative experiments. Firstly, 50 mg/L mixed standards of DAS, DADS, and DATS were separated using the mobile phase comprised of (0.10% TFA in 85%ACN–15%DW):(0.10% TFA solution in DW) (90:10) at a 1.0 mL/min flow rate, 220 nm wavelength and 8.0 min run time. The initial column tested for the chromatographic separation of the analytes was Zorbax ODS (250 mm, 4.6 mm, 5μm). The retention times of the analytes were determined by running the standards separately and as a mixture through the chromatographic system. The peaks had poor resolution; thus, it was decided that the column was not suitable for proper separation of the analytes. A different C18 column (Phenomenex Nucleosil: 250 mm, 4 mm, 5μm) was therefore tested by running three different concentrations (20, 50, and 100 mg/L mix standard solutions) of the analytes through the column to the detector. A linear increase in peak area was obtained with increasing concentrations of the analytes. Even though the retention times of the OSCs’ peaks were verified, peak tailings were observed for the tested column, and this affected the resolution of separation. In order to shorten the retention times of the analytes and obtain sharp peaks, a relatively new Phenomenex column (Phenomenex Nucleosil 250 mm, 4 mm, 5 mm) was also tested. This column produced better peak resolutions and was further optimized to obtain sharp and narrow peaks. Thus, the Phenomenex column was selected for chromatographic elution of the target analytes.

The mobile phase was initially optimized to obtain a better peak shape. The duration of analyte retention is achieved through modulation of the mobile phase composition [23]. Different ratios of acetonitrile:water and TFA:water mixtures were tested, and the narrowest peaks were obtained using 0.1% TFA in 85% ACN:0.1% TFA solution (90:10) mixture. The composition of the mobile phase was optimized by testing 80:20, 70:30, 60:40 and 90:10 (ACN: TFA, v/v) ratios. An increase in the proportion of water in the mobile phase resulted in an increase in retention, while a higher concentration of the organic solvent led to a decrease in retention. As a result of the optimization experiment, all three analytes were retained more with a decrease in the percentage of ACN in the mobile phase. The outcome was characterized by broader peaks, which exhibited a linear correlation with the reduction in ACN quantity. A reduction in resolution was observed, particularly for DAS and DADS analytes. The mobile phase ratio of 90:10 was found to be suitable for achieving a high peak resolution and maintaining an optimal chromatographic separation time, thereby facilitating the method’s applicability. The 90:10 mobile phase ratio yielded the narrowest and sharpest peak shapes, and thus, it was selected as optimum ratio. The efficacy of a high-performance liquid chromatography (HPLC) technique utilizing ultraviolet (UV) detection is contingent upon the appropriate choice of detection wavelength. This selection can be ascertained by capturing superimposed UV spectra [24]. The wavelength range between 185 and 300 nm was scanned using standard solutions of the analytes. To achieve this objective, standard solutions of the analytes were individually determined with the UV-Vis spectrometer, and the maximum wavelength was ascertained for each analyte. In the measurements, three different concentrations of the analyte were prepared, and the solutions were measured in the system, where the maximum wavelength for each analyte was noted. Since the highest absorbance for the analytes were observed around 220 nm, this wavelength was selected as the optimum wavelength.

In the context of HPLC applications, expediting the separation of compounds is a preferred choice and this can be achieved by setting flow rates above specific thresholds [25]. Variations in mobile phase flow rate affect the interaction of analytes with the stationary phase and the rate of elution through the column. The optimization experiments were continued with the flow rate of the mobile phase, and the variables tested were 0.80, 1.0, 1.1, and 1.2 mL/min. The 0.8 mL/min flow rate resulted in extended retention of the analytes in the column, and this caused a significant peak broadening. The resolution determined for DAS and DADS was notably low (k^’^ ≤ 1.0). Nonetheless, the reduction in retention times as flow rate increased resulted in a decrease in analyte resolution. Furthermore, the chromatogram obtained for the three analytes exhibited a relatively low resolution. The optimal sample flow rate for the chromatographic system was determined to be 1.0 mL/min, as it provided the highest resolution in the given situation. Injection volumes including 10, 20, 30, 40, and 50 μL were tested to ascertain the effect of different volumes on the peak shapes of the analytes. As anticipated, larger peak heights/areas were noted with the increase in injection volume owing to the augmented quantity of the analytes. Nevertheless, augmentation in the peak height/area observed at concentrations exceeding 20 μL did not exhibit a linear correlation but resulted in peak tailings of the analytes. Considering all these, 20 μL was selected as the optimum injection volume because it produced a relatively transient peak shape for the analytes.

The determined optimum conditions were examined in the AGREEprep software [26]. According to the results, the method demonstrated high acceptance in green approach, scoring 0.65 in the evaluation.**3.2 Analytical figures of merit**

**Figure 2:**
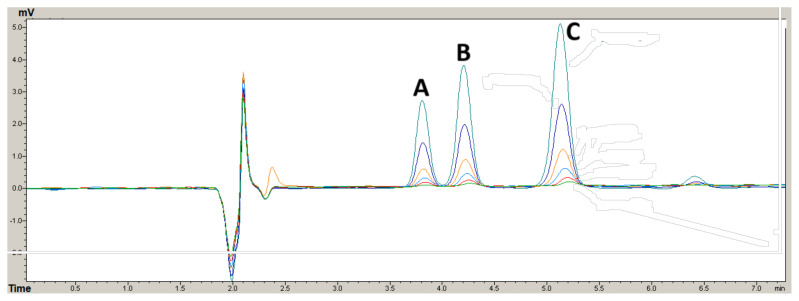
The overlay chromatogram of the OSC compounds at different concentrations in the linear working range of the developed method, (A) DAS, (B) DADS, and (C) DATS.

The optimum experimental conditions for the developed method were determined, and under these conditions, the analytical performance of the system was evaluated using mixed standard solutions of the analytes. The method recorded coefficient of determination values ranging from 0.9997 to 1.00, and detection limits calculated for the analytes were in a low range of 0.09 to 0.17 mg/kg. Overlay chromatograms for mix standard solutions prepared at different concentrations can be seen in Figure 2. With the analyte peaks obtained from 20 mg/kg mixed standard solution, the selectivity factor (α) was calculated and found to be 1.24 and 1.43 for DAS-DADS and DADS-DATS, respectively. In calculating the limits of detection (LOD) and quantification (LOQ), it was ensured that the lowest concentrations in the calibration plots of the analytes had signal/noise ratios higher than three (3). The percent relative standard deviation (%RSD) value was calculated for each analyte at this concentration (n = 6) and recorded as 5.9, 11.5, and 11.5% for DAS, DADS, and DATS, respectively. The standard deviation (SD) of the lowest concentration of the calibration plot was determined through six replicate measurements. This value was then used to calculate the limits of the method as expressed below [27–29]:


$$\begin{array}{l} \text{LOD}=3\times \text{SD}/\text{slope of calibration plot},\\ \text{LOQ}=10\times \text{SD}/\text{slope of calibration plot.} \end{array}$$

As indicated in Table 1, the analytical figures of merit obtained for the developed system are comparable with those of other HPLC-based methods reported in the literature. Additionally, some gas chromatography-mass spectrometry systems have been employed to determine organosulfur compounds in garlic samples [30].

### 3.2. Real sample application

**Figure 3:**
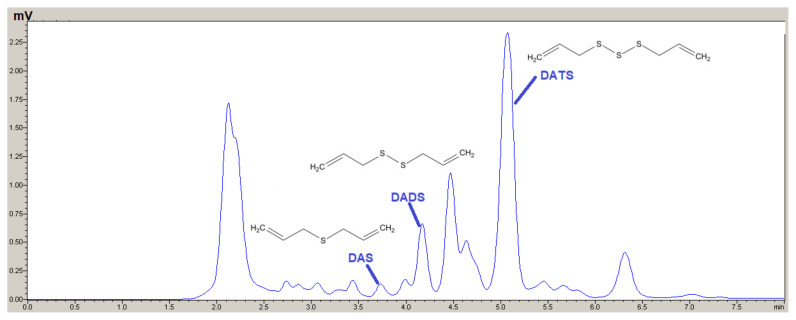
A chromatogram of sample 1 obtained after extraction procedure for oil-soluble OCS compounds.

To validate the applicability of the developed method, real sample analyses were performed with garlic samples. Specifically, four different garlic samples were obtained from the Kastamonu Taşköprü region, and the extraction procedure described in the methods section was applied to three replicates of each sample under the optimum conditions. The samples were analyzed for their oil-soluble and water-soluble organosulfur compounds. Analytical signals for analytes in the oil-soluble extracts were obtained (Figure 3), but no signal was observed for the water-soluble extracts. The concentrations of the analytes in the extracted garlic samples were calculated and are summarized in Table 2.

None of the allyl sulfur compounds were detected in the water-soluble extracts of the garlic samples. To evaluate the accuracy of the developed method for the water-soluble components of the samples, four Kastamonu Taşköprü garlic samples were spiked with 10 mg/kg of the allyl sulfur compounds for recovery experiments using the optimum RP-HPLC-UV conditions. Calibration plots developed with mixed standard solutions of the analytes were used to quantify the analytes in the spiked garlic samples. Overlay chromatograms of the spiked samples and a 10 mg/kg mixed standard solution showed that there was no shift in retention times of the analytes, which could have resulted from matrix effects. This validated the ability of the developed chromatographic method to be used for accurate and precise determination of the analytes irrespective of the sample matrix. The recovery values for the spiked samples were calculated in the range of 39.0% to 90.9% (Table 3). The low relative standard deviations (<20%) obtained for all spiked samples indicated appreciable repeatability for replicate extractions and measurement.

## 4. Conclusion

In this study, a simple and sensitive analytical method was developed for the determination of DAS, DADS, and DATS with relatively low detection limits in a garlic matrix by RP-HPLC-UV system. The developed chromatographic method allowed distinct separation of the allyl sulfide compounds from each other with good resolution and a short analysis period. To enhance the detection power of the developed system, all instrumental and experimental conditions were optimized. The applicability and accuracy of the method was verified using Kastamonu Taşköprü garlic as a real sample matrix. Oil-soluble extracts containing the analytes in the range of 8.0 to 356.6 mg/kg were determined in the Kastamonu Taşköprü garlic samples. None of the allyl sulfur compounds was detected in the water-soluble extracts of the garlic samples. The procedure for the water-soluble extracts was validated by performing spike recovery experiments with a fortified concentration of 10 mg/kg. The recovery results ranged between 39.9% and 90.9%. The results indicated that the method can be accurately and precisely used to quantify the allyl sulfur compounds in the garlic matrix, depending on the water-soluble interferent content. Each sample has a different matrix; hence, the recovery values varied among them. Matrix matching could be applied to increase efficiency.

## Figures and Tables

**Table 1:** The comparison between system analytical performance values of the developed method and other studies in the literature.

Method	Analyte	LOD	LOQ	LWR	Sample	Ref.

HPLC-UV	DAS	15 mg kg^−1^	36 mg kg^−1^	0.002–0.005 mg mL^−1^	Italian garlic steam	[31]
	DADS	7 mg kg^−1^	15 mg kg^−1^	0.001–0.097 mg mL^−1^		
	DATS	4 mg kg^−1^	9 mg kg^−1^	0.0005–0.096 mg mL^−1^		

DLLME-HPLC-UV	DAS	1.05 mg mL^−1^	1.5 mg mL^−1^	10–200 mg mL^−1^	Argentina garlic	[32]
	DADS	0.16 mg mL^−1^	1.13 mg mL^−1^	10–200 mg mL^−1^		

RP-HPLC-UV	DAS	2.41 mg mL^−1^	7.29 mg mL^−1^	4.40–353.30 mg mL^−1^	Korean garlic	[33]
	DADS	1.73 mg mL^−1^	5.25 mg mL^−1^	10.10–807.00 mg mL^−1^		
	DATS	1.22 mg mL^−1^	3.68 mg mL^−1^	8.80–700.00 mg mL^−1^		

SPME-HPLC-UV	DAS	31 mg kg^−1^		89–357 mg kg^−1^	Argentina garlic	[34]
	DADS	14 mg kg^−1^	-	68–272 mg kg^−1^		
	DATS	25 mg kg^−1^		100–400 mg kg^−1^		

RP-HPLC-UV	DAS	0.12 mg kg^−1^	0.42 mg kg^−1^	0.503–100.00 mg kg^−1^	Kastamonu Taşköprü garlic	This study
	DADS	0.17 mg kg^−1^	0.57 mg kg^−1^	0.502–101.01 mg kg^−1^		
	DATS	0.09 mg kg^−1^	0.31 mg kg^−1^	0.266–100.82 mg kg^−1^		

**Table 2:** Analyte concentrations in extracted garlic samples for oil- and water-soluble extractions.

Number of sample	Analyte	Found concentration (mg kg^−1^)	
		Oil-soluble extractions	Water-soluble extractions
1	DAS	32.5 ± 1.3	N.D.*
	DADS	67.3 ± 5.5	N.D.
	DATS	356.6 ± 26.3	N.D.
2	DAS	14.5 ± 3.7	N.D.
	DADS	34.8 ± 3.3	N.D.
	DATS	207.0 ± 22.3	N.D.
3	DAS	14.3 ± 3.2	N.D.
	DADS	26.8 ± 3.8	N.D.
	DATS	63.7 ± 1.3	N.D.
4	DAS	8.0 ± 1.2	N.D.
	DADS	20.4 ± 0.1	N.D.
	DATS	60.7 ± 7.0	N.D.

* N.D.: not detected.

**Table 3:** Recovery results for spiking experiments.

Number of sample	Analyte	Spike concentration (mg kg^−1^)	Found concentration(mg kg^−1^)	Recovery% ± SD
1	DAS	10.01	7.7 ± 0.8	71.3 ± 12.7
	DADS	10.11	7.6 ± 0.6	81.8 ± 9.1
	DATS	10.09	8.8 ± 0.4	90.9 ± 5.7
2	DAS	10.01	5.9 ± 0.2	56.6 ± 5.8
	DADS	10.11	6.8 ± 0.2	74.5 ± 4.5
	DATS	10.09	8.2 ± 0.5	84.7 ± 7.2
3	DAS	10.01	4.8 ± 0.5	47.7 ± 18.0
	DADS	10.11	5.9 ± 0.3	65.3 ± 6.8
	DATS	10.09	8.0 ± 1.1	82.3 ± 15.9
4	DAS	10.01	3.8 ± 0.3	39.0 ± 14.2
	DADS	10.11	5.6 ± 0.4	64.7 ± 2.9
	DATS	10.09	8.0 ± 0.4	81.9 ± 5.0
